# Workload and Enjoyment Perception in Small-Sided Soccer Games: A Systematic Review of Studies in Untrained Children and Adolescents

**DOI:** 10.1177/19417381251385590

**Published:** 2025-10-31

**Authors:** Nicolás Gómez-Álvarez, Leonel Federico-Tuccelli, Paula SanMartín-Godoy, Mario Vieyra-Fuenzalida, Felipe Hermosilla-Palma, Tomás Reyes-Amigo, José Oliveira, Hélder Fonseca

**Affiliations:** † Centre for Research, Education, Innovation and Intervention in Sport, Faculty of Sport of the University of Porto, Porto, Portugal; ‡ Research Nucleus in Human Motricity Sciences (NICMOH), Universidad Adventista de Chile, Chillán, Chile; § Pedagogía en Educación Física, Facultad de Educación, Universidad Autónoma de Chile, Talca, Chile; ‖ Physical Activity Sciences Observatory (OCAF), Department of Physical Activity Sciences, Universidad de Playa Ancha, Valparaíso, Chile; ¶ Research Centre in Physical Activity, Health, and Leisure (CIAFEL), Faculty of Sport, University of Porto, Porto, Portugal; # Laboratory for Integrative and Translational Research in Population Health (ITR), Porto, Portugal

**Keywords:** exercise, physical activity, sports, training

## Abstract

**Context:**

Small-sided soccer games (SSSG) have been proposed as a strategy to promote the health of children and adolescents. Understanding training loads has a strong influence on program effectiveness.

**Objective:**

This systematic review aimed to describe the training load and perception of enjoyment during SSSG in untrained children and adolescents.

**Data Sources:**

A systematic search on PubMed, Web of Science, Scopus, and Scielo databases was performed.

**Study Selection:**

Experimental or observational studies conducted in untrained children or adolescents (6 to 18 years) that analyzed internal loads, external loads, or perceived enjoyment during 1 or more SSSG were included.

**Study Design:**

Systematic review following PRISMA guidelines.

**Level of Evidence:**

Level 3.

**Data Extraction:**

Information on publication type, participants, SSSG characteristics, and main results of the internal and external load and perceived enjoyment was extracted.

**Results:**

A total of 15 articles (n = 539 participants, aged 8-18 years) were included. Twenty-one SSSG designs were identified, and the format used most was 3v3. Heartrate (HR) (range 71%-88% of maximum HR) and rate of perceived exertion (range 3-7 of 10 or 12-15 of 20) were the primary measures of internal load, while distance traveled, average speed, and number of accelerations were used to assess external load. Perceived enjoyment was reported using mainly the 16-item PACES or 18-item PACES scale (59.14 or 88.67, respectively). The structural aspects of the game were evaluated in 6 studies, suggesting that the number of players, pitch size, man-marking, and restricting the type of locomotion may affect workload. Contextual factors, such as verbal motivation or sex, can also influence workload.

**Conclusion:**

SSSG is associated with moderate-to-vigorous intensity exercise and high enjoyment perception. Workload can be affected by structural (number of players, pitch size, man-marking) and contextual factors (verbal encouragement, sex composition), although evidence is still limited.

The World Health Organization (WHO) recommends that children and adolescents perform at least 60 minutes of moderate-to-vigorous physical activity daily and engage in vigorous-intensity and strengthening activities at least 3 days per week.^3^ However, adherence to these guidelines remains suboptimal, highlighting the need to optimize interventions to promote higher levels of physical activity adherence.^11,15^ Consequently, crafting engaging and motivating interventions could be instrumental in increasing children and adolescents’ adherence to physical activity guidelines.^27,48^

Small-sided soccer games (SSSG) have emerged as a promising approach due to their playful, social, and motivating nature.^12,37,49^ These games encourage intermittent high-intensity efforts, which have been associated with benefits such as improved cardiorespiratory fitness.^14^ Moreover, the enjoyment reported by participants during the SSSG could promote adherence and compliance to interventions that include this type of activity.^9,41^ However, studies evaluating SSSG interventions have reported mixed results regarding physical fitness or health outcomes,^7,17,46^ potentially due to the variability in training loads.

Training loads during SSSG are determined by structural design, training regimen, and several contextual factors.^5,21^ Studies in athletes have shown that game format, pitch configuration, restriction of actions, training regime, and contextual factors, all affect SSSG load.^2,5,40^ Adjusting these parameters is particularly important for untrained children and adolescents, as they lack specific or systematic training and often exhibit greater variability in physical fitness levels and soccer-related skills. For instance, SSSG-based programs have employed diverse structural configurations, including player numbers ranging from 2vs2 to 7vs7 and playing field sizes varying from 40 m^2^ to 100 m^2^ per player.^17,46^ These variations can result in distinct functional adaptations and training outcome,^21,22,24^ highlighting the need to customize SSSG programs to address the specific requirements of this population.

Studies on SSSG have shown that these programs typically elicit moderate-to-vigorous intensities, ranging from 71% to 85% of maximum heartrate (HR).^7,46^ Variations in perceived enjoyment - a key factor influencing program adherence - have also been observed and may be affected by individual participant characteristics.^25^ These findings highlight the necessity for a more nuanced understanding of the interplay between workload and enjoyment in SSSG intervention to maximize their effectiveness and accessibility.

Therefore, intervention programs focused on the health of children and adolescents who take part in SSSG require knowledge of the strategies used to increase or decrease the workload and enjoyment of the SSSG to optimize the training prescription and increase program adherence and fidelity. This systematic review aimed to describe training load and perceived enjoyment during SSSG in untrained children and adolescents. The secondary objective was to determine the effects of the structural and contextual variables determining internal and external SSSG loads. Organizing and summarizing this information could optimize the implementation of SSSG to improve the fitness and health of children and adolescents.

## Methods

This systematic review followed the recommendations and criteria established by the Preferred Reporting Items for Systematic Reviews and Meta-analyses (PRISMA) Reporting Guidelines.^31^

### Eligibility Criteria

Population characteristics, intervention type, type of outcome, and study design were used to determine the eligibility criteria. Studies that met the following inclusion and exclusion criteria were included:

(1) Population: children or adolescents (6-18 years) who were untrained (without participation in regular, systematic or structured soccer-specific training programs aimed at improving performance or competing at a formal level),^32^ or participated in noncompetitive sports training. Studies conducted on trained participants with ≥3 practices per week or those participating in competitive sports were excluded.
(2) Intervention: studies including ≥1 SSSG variations were included. Studies that did not include SSSG nor described the SSSG intervention were excluded.
(3) Results: studies that analyzed acute responses during or immediately after SSSG related to internal load (HR, rate of perceived exertion [RPE], or lactate), external load (distance traveled, speed, or acceleration), or perceived enjoyment were included. Perceived enjoyment was defined as a positively valued emotion directed toward SSSG activities, associated with feelings such as pleasure, joy, and fun. Prospective studies evaluating the long-term effects of an SSSG-based intervention and studies reporting measures of workload or enjoyment corresponding to the complete training session (e.g., including warm-up or cool-down) were excluded.
(4) Study design: experimental or observational studies were included. Systematic reviews, narrative reviews, letters to the Editor, or studies on experimental animals were excluded.

### Literature Search Strategy

A literature search was conducted in November 2024 using 4 electronic databases: PubMed, Web of Science, Scopus, and Scielo. Key search terms were included and combined using the “AND,” “OR” Boolean operators: children OR childhood OR adolescent OR youth OR young AND (small-sided games OR small-sided soccer games OR SSG OR SSSG) AND (soccer OR Football) AND (workload OR response OR demands OR “acute effects” OR load OR physiological OR intensity OR “heart rate” OR lactate OR velocity OR acceleration OR deceleration OR enjoyment). In addition, a manual search of the reference lists of the relevant studies identified after the initial database search was performed.

### Selection of Studies

All records identified in the databases or searched manually were exported to EndNote (Version 20.6; Clarivate Analytics), where duplicates were removed. All records were then exported to Rayan software for the study selection process.^30^ Three reviewers independently screened the studies in 2 stages: first, by evaluating titles and abstracts, and then by analyzing the full texts of potentially eligible studies based on the predefined eligibility criteria. In cases of disagreement, all 3 reviewers, along with the principal investigator, discussed the discrepancies and reached a consensus through deliberation.

### Data Extraction and Synthesis

Two researchers independently extracted the data from reports that met the eligibility criteria. A pre-established data extraction spreadsheet was used to extract information regarding publication type, participants, SSSG characteristics, and main results of the internal and external load and perceived enjoyment. The following general information was also collected: authors, year of publication, country, research purpose, and total number of participants.

For participant characteristics, age and sex were recorded. Regarding SSSG attributes, data were extracted on pitch size, number of players, rules, and game setup (e.g., number of sets, duration per set, and rest intervals). Measures of internal load (e.g., HR, RPE, blood lactate), external load (eg, distance traveled, speed, accelerations), and perceived enjoyment during SSSG were also recorded. In addition, when studies reported results for distinct subgroups within the same research (eg, different SSSG configurations or participant classifications), data for each subgroup were extracted separately.

For the synthesis of information, means and standard deviations were obtained for each of the internal or external load measures reported, including each of the subgroups generated within each study. When the standard error of the mean was reported, the standard deviation was calculated using the formula SD = SEM × √n. When information was only available in graphs,^16,28,45^ the WebPlotDigitizer (https://automeris.io/WebPlotDigitizer) was used. This information provided a descriptive summary for each internal and external load measure, including the mean, standard deviation, minimum, maximum, mode, and median. In addition, when a study compared different SSSG types, the reported statistical test results were used to identify differences between SSSG models.

### Quality Assessment

To assess the study’s quality, a validated risk of bias form specific to this systematic review topic was used.^39^ The instrument is composed of 16 items that are scored with 0 (no) or 1 (yes), with items 6 and 13 also having an option of nonapplicable (NA). Questions were related to the clarity of study purpose (item 1), relevance of background literature (item 2), appropriateness of the study design (item 3), sample included (items 4 and 5), informed consent (item 6), outcome measures (items 7 and 8), methods description (item 9), significance of the results (item 10), analysis (item 11), practical importance (item 12), description of dropouts (item 13), conclusions (item 14), practical implications (item 15), and limitations (item 16). The overall score was calculated as the sum of the items answered, and a percentage score was used to classify the results according to methodological quality. Reports were classified as^39^: (1) low methodological quality (≤50%), (2) good methodological quality (between 51% and 75%), and (3) excellent methodological quality (>75%).

## Results

### Study Selection

Figure 1 shows detailed information on the study selection process. The search identified 979 studies, of which 398 were eliminated as duplicates. After reviewing the titles and abstracts and assessing the eligibility criteria, 13 studies were included for qualitative analysis.^1,10,16,18,19,26,28,29,33,36,38,43,45^ Two additional reports were identified and included after a manual search of references.^4,13^

**Figure 1.**
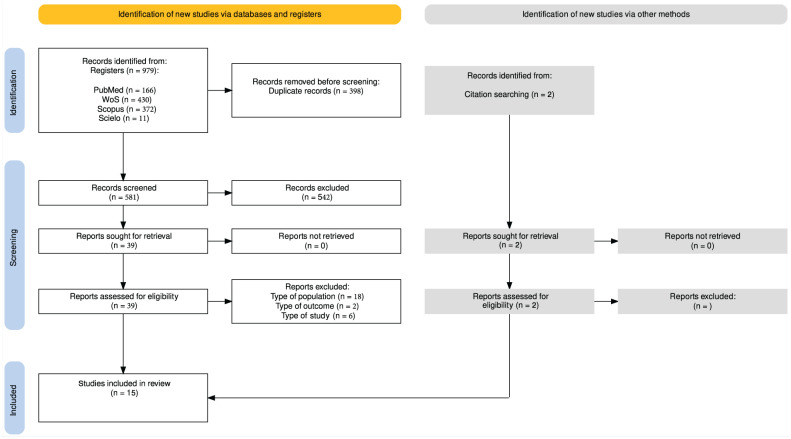
PRISMA flow diagram.

### Quality of the Studies

The methodological quality assessment results showed that studies had good or excellent methodological quality, with scores between 75% and 93.8%. The item where most deficiencies were identified was in sample size calculation (item 5), together with statement of study limitations (item 16), and reporting of practical implications (item 12) (Table 1).

**Table 1.** Quality assessment of the studies

Study	Item																Score	
	1	2	3	4	5	6	7	8	9	10	11	12	13	14	15	16	Raw	%
Castagna et al^4^	1	1	1	1	0	1	1	1	1	1	1	1	NA	1	1	0	13	81.3
Randers et al^36^	1	1	1	1	0	1	1	1	1	1	1	0	NA	1	1	0	12	75.0
Toh el al^45^	1	1	1	1	0	1	1	1	1	1	1	0	NA	1	1	0	12	75.0
Ngo et al^29^	1	1	1	1	0	1	1	1	1	1	1	0	NA	1	1	1	13	81.3
Bendiksen et al^1^	1	1	1	1	0	1	1	1	1	1	1	1	NA	1	1	1	14	87.5
Martins Coutinho et al^28^	1	1	1	1	0	1	1	1	1	1	1	1	NA	1	1	0	13	81.3
Hammami et al^16^	1	1	1	1	0	1	1	1	1	1	1	1	NA	1	1	1	14	87.5
Hammami et al^18^	1	1	1	1	0	1	1	1	1	1	1	1	NA	1	1	1	14	87.5
Póvoas et al^33^	1	1	1	1	1	1	1	1	1	1	1	1	NA	1	1	1	15	93.8
Smallcombe et al^43^	1	1	1	1	0	1	1	1	1	1	1	0	NA	1	1	1	13	81.3
Evangelio et al^10^	1	1	1	1	0	1	1	1	1	1	1	1	NA	1	1	1	14	87.5
Lind et al^26^	1	1	1	1	0	1	1	1	1	1	1	1	NA	1	1	1	14	87.5
Gómez-Álvarez et al^13^	1	1	1	1	0	1	1	1	1	1	1	0	NA	1	1	1	13	81.3
Sahli et al^38^	1	1	1	1	0	1	1	1	1	1	1	1	Na	1	1	1	14	87.5
Hammami et al^19^	1	1	1	1	0	1	1	1	1	1	1	1	Na	1	1	1	14	87.5

1, clarity of study purpose; 2, relevance of background literature; 3, appropriateness of the study design; 4, detailed description of the sample; 5, justification of sample size; 6, informed consent; 7, reliable outcome measures; 8, valid outcome measures; 9, methods description; 10, significance of the results; 11, method of analysis; 12, practical importance; 13, description of dropouts; 14, conclusions; 15, practical implications; 16, limitations; NA, nonapplicable.

### Characteristics of the Studies and Participants

All pooled studies were published between 2007 and 2023. Of these, 4 studies were conducted in Tunisia,^1,16,18,19,38^ 3 in Denmark,^1,26,36^ 2 in Portugal,^28,33^ and 1 each in China,^29^ Australia,^45^ Italy,^4^ Spain,^10^ Chile,^13^ and England (Table 2).^43^

**Table 2.** General characteristics of the studies included in the systematic review

Author, country	SSSG participants	Type of comparison of SSSG	SSSG formats	Work duration, min (S/R/P)	Pitch dimension, length × width, m ( ApPm^2^)	Rules	Workload measurements and enjoyment perception
Castagna et al,^4^ Italy	15 (M); 16.7 ± 1.2 years	-	5v5	30	30 × 15 (45)	Not verbally encouraged Immediate ball replacement With goalkeeper	HR (bpm - HR zone) RPE
Randers et al,^36^ Denmark	75 (M-F); 39 of 9 years; 36 of 12 years	Age/pitch dimension	3v3	66 (4/15/2)	C9: 9 × 7 (10.5)	Extra balls No specific coaching instruction	HR (%HRM; bpm; HR zone)
					C12: 20 × 13 (43)		
Toh et al,^45^ Australia	12 (M) 10.7 ± 1.2 years	Pitch dimension	3v3	35 (2/15/5)	6.1 × 13.4 (13)	No offside rules Without goalkeeper substitution or sideline encouragement	HR (%HRM, HR zones) RPE Enjoyment perception (PACES) Accelerometry
					9 × 18 (27)		
					14.2 × 26.5 (62)		
Ngo et al,^29^ China	12 (M); 16 ± 0.7 years	Defensive rule manipulation (MM vs NMM)	3v3	20 (3/4/4)	18 × 25 (75)	MM and NMM Verbal encouragement	HR (mean %HRR) RPE
Bendiksen et al,^1^ Denmark	93 (M-F); 8-9 years	Characteristics of the participants (sex, sport participation and BMI)	3v3	33 (2/15/3)	20 × 40 (133)	Without goalkeepers. All players had to pass the midway line for the team to score	HR (bpm; mean %HRM; HR zone) RPE
Coutinho et al,^28^ Portugal	9 (M); 13.3 ± 0.7 years	Players numbers and goals numbers	3v3	4	20 × 15 (50)	1 or 2 targets 1 or 2 goalkeepers were used 1 team performed offensive actions and the other only defensive. Each attack was initiated on the end line	HR (HR zone) RPE
			4v4		20 × 20 (50)		
Hammami et al,^16^ Tunisia	12 (M); 15.8 ± 0.6 years	-	3v3	13 (3/3/2)	Increased pitch size 20 × 12 (40) 25 × 15 (62) 30 × 18 (90)	Motivated to maintain high intensity	HR (bpm; RHR) RPE Enjoyment perception
Hammami et al,^18^ Tunisia	10 (M); 15.8 ± 0.6 years	-	3v3	13 (3/3/2)	Increased pitch size 20 × 12 (40) 25 × 15 (62) 30 × 18 (90)	Motivated to maintain high intensity	HR (bpm) RPE
Póvoas et al,^33^ Portugal	134 (M-F) 12-16 years	Sex and player number	2v2	32 (2/15/2)	16 × 12 (50)	No goalkeepers Offside rule was not applied All players had to pass the midway line for the team to score	HR (%HRM; HR zone; RPE) Enjoyment perception
			4v4		23 × 17 (50)		
			12v12		40 × 30 (50)		
Smallcombe et al,^43^ England	15 (M); 12.6 ± 0.5 years	-	5v5	48 (6/8/ND)	44 × 22 (96)	Goalkeeper rotation every 2 minutes	HR (bpm) RPE Time-motion characteristics (distance; mean velocity)
Evangelio et al,^10^ Spain	57 (M-F): 10.44 ± 1.30 years	Age	3v3	5	18 × 14 (42)	4 goals and could score in any of them. Score only when all members of a team are within the shooting zone and control the ball ≥1 time	HR (bpm) Distance travelled Number of accelerations
Lind et al,^26^ Denmark	27 (M-F) 11-12 years	Restriction in the type of locomotion. SRF vs SWF	3v3	25 (2/10/5)	6 × 16 (16)	All players had to pass the midway line for the team to score	HR (HR zone) Enjoyment perception
Gómez-Álvarez et al,^13^ Chile	12 (M) 11-14 years	Continuous vs fractionated regime	3v3	16 (1 × 16) 16 (4 × 4 × 2)	18 × 9 (27)	All players had to pass the midway line for the team to score	HR (bpm, %HRM) Enjoyment perception
Sahli et al,^38^ Tunisia	16 (M); 17.37 ± 0.48 years	Verbal encouragement	4v4	25 (4/4/3)	35 × 25 (109)	Limit of 2 ball touches per contact	HR (mean %HRM) Lactate RPE Enjoyment perception
Hammami et al,^19^ Tunisia	52 (F); 16.5 ± 0.4 years	Verbal Encouragement	5v5	10	35 × 25 (88)	Minimum of 3 ball touches to score	HR (mean bpm)

ApPm^2^, area per player in square meters; bpm, beats per minute; BMI, body mass index; C9, 9-year-olds; C12, 12-year-olds; F, female; HR, heartrate; HRM, maximal heartrate; M, male; MM, with man marking; NMM, without man marking; PACES, physical activity enjoyment scale; RHR, resting heartrate; RPE, rate of perceived exertion; S/R/P, sets/repetitions/rest; SSSG, small-sided soccer game; SRF, real soccer games; SWF, walking soccer game.

This systematic review is based on data from 539 children and adolescents who participated in the included studies, with sample sizes ranging from 9 to 134 participants. Nine studies were conducted with <20 participants and 6 with >20. Regarding participant’s sex, 9 studies included only boys,^4,13,16,18,28,29,38,43,45^ 5 included both boys and girls,^1,10,26,33,36^ and 1 included only girls.^19^

Participants age was between 8 and 18 years old, with 5 reports including children between 8 and 12 years of age,^1,10,26,36,45^ while 10 included adolescents between 12 and 17 years of age.^4,13,16,18,19,28,29,33,38,43^

### SSSG Characteristics

The general characteristics of the SSSG are presented in Table 3. From the 15 studies analyzed, 21 different SSSG designs were identified.

**Table 3.** Summary of the design characteristics of SSSG structures

	k/g	Mean ± SD	Median	Mode	Minimum-maximum
Format					
Player numbers	15/21	3.3 ± 0.8	3	3	2-5
Pitch configuration					
ApP, m^2^	13/19	53.3 ± 34	50	50	10-133
Length, m	13/19	22.8 ± 18	18	18	9-44
Width, m	13/19	14.5 ± 6.2	14	20	6-25
Regimen organization					
Bouts, n	12/18	3.0 ± 1.3	3	2	1-6
Bout duration, minutes	12/18	10.0 ± 5.5	12.5	15	3-15
Rest, minutes	11/16	3.1 ± 1.3	3	2	2-5
SSG duration, minutes	15/21	38.7 ± 16.0	25	35	5-66

ApP: area per player; g, number of subgroups; k, number of studies; SSSG, small-sided soccer game.

Regarding the type of format and court configuration, 3v3 was used in 13 games with pitch sizes between 10.5 m^2^ and 133 m^2^ per player.^1,10,13,16,18,26,28,29,36,45^ The 4v4 format was used in 3 studies with pitch sizes between 50 m^2^ to 109 m^2^ per player.^28,33,38^ The 5v5 format was also used in 3 studies with pitch sizes between 45 m^2^ and 96 m^2^ per player.^4,19,43^ A single research employed the 2v2 format with a pitch size of 50 m^2^ per player; 12 studies used fractional game setup, while 4 studies used continuous game.

Some studies also described constraints and modifications to SSSG or special rules during the game. One study altered the scoring method by including variations such as scoring with 1 or 2 soccer goals,^28^ while 2 studies examined the presence or absence of goalkeepers.^4,43^ Other rules modifications included the exclusion of the offside rule,^33,45^ requiring the entire team to move to the opponent’s side of the pitch before scoring a goal,^13,33^ limiting the number of touches each player could make with the ball,^38^ and altering the type of marking (eg, man-to-man).^29^ In addition, 5 studies reported the use of verbal encouragement to maintain high-intensity.^16,18,19,29,38^

### Main Results

Among the 15 studies included in this systematic review, a total of 31 subgroups reported ≥1 workload variable or enjoyment perception measures. Six studies included subgroup analyses according to structural adjustment or regimen type,^13,26,28,29,33,45^ and 6 studies included subgroup analyses according to contextual factors.^1,10,19,33,36,38^

Internal load was monitored mainly by physiological responses (HR and capillary blood lactate concentration) and RPE to SSSG. External loads were quantified using time-motion measurements and accelerometer-derived measurements. Questionnaires assessed perceived enjoyment. Table 4 presents a descriptive summary of the main internal and external load and enjoyment perception measures reported.

**Table 4.** Synthesis of the main internal and external workload measures reported during SSSG

Measure	Internal load			External load				
	k/g	Mean ± SD (median)	Minimum-maximum	Measure		k/g	Mean ± SD (median)	Minimum-maximum
HR response				Time-motion measurements				
bpm	10/31	158 ± 9 (157)	145-178	Total distance		1/1	3.6 ± 0.4	
%HRM	6/30	77.9 ± 4.5 (77)	71.0-88.6	Mean velocity		1/1	4.4 ± 0.5	
%HRR	¼	78.2 ± 2.7 (6.6)	75.7-80.5	Maximal velocity		1/1	9.7 ± 3.3	
%T 70-80%HRM	2/5	29.9 ± 6.1 (32.5)	21.7-35.5	%T at < 0.1 m/s		1/1	10.1	
%T 70-85%HRM	1/1	39.7±19.9	-	%T at 0.1-0.8 m/s		1/1	30.8	
%T >80 HRM	2/11	40.4 ± 5.8 (38)	34.0-55.0	%T at >0.8-2.2 m/s		1/1	43.2	
%T 80-90%HRM	2/5	31.9 ± 2.9 (31.1)	29.3-36.9	%T at >2.2-3.6 m/s		1/1	12	
%T 85-90%HRM	¼	15.6 ± 9.1 (16.0)	5.3-25.3	%T at >3.6-5.0 m/s		1/1	3.5	
%T >85%HRM	1/1	50.9 ±26.1	-	%T at >5.0 m/s		1/1	0.2	
%T 90-100%HRM	2/11	22.9 ± 17.2 (13)	10.0-59.5	Accelerometer measurement				
%T 90-95%HRM	2/5	12.7 ± 5.4 (12.9)	3.9-17.6	Counts per minute		1/3	2614.0 ± 180.3 (955.0)	2436.9-2797.4
%T 95-100%HRM	2/5	4.9 ± 2.6 (5.9)	0.8-17.6	N-Acc -1.99; -1.00		1/1	18 ± 7.8	-
%T 70-80%HRR	1/1	23.7 ± 4.9	-	N-Acc -0.99; 0.50		1/1	51.3 ± 14.4	-
%T 80-90 %HRR	1/1	35.9 ± 6.9	-	N-Acc 0.50; 0.99		1/1	51 ± 14.3	-
%T 90-100 %HRR	1/1	15.8 ± 7.1	-	N-Acc 1; 1.99		1/1	15.9 ± 7.3	-
Metabolic response								
Lactate	1/2	4.03 ± 0.57	3.62-4.43					
Rate Perception Effort								
Borg 0-10	5/20	5.3 ± 1.3 (5.5)	3.27-7.4					
Borg 6-20	2/7	13.8 ± 1.1(14.1)	12.1-15.2					
VAS 0-10	1/1	2.4 ± 0.3	-					
OMNI 0-10	1/1	5 ± 2	-					
	Perceived enjoyment							
PACES-16*^a^*	3/6	50.5 ± 14.5 (53.5)	33.2-65.9					
PACES-18*^a^*	2/4	88.6 ± 11.9 (91.5)	75.6-98.9					
FUN Scale 0-10*^a^*	1/12	6.0 ± 0.9 (4.9)	4.4-7.3					

bpm, beats per minutes; g, number of subgroups; HR, heartrate; %HRM, percentage of maximum HR; %HRR, percentage of the reserve HR; k, number of studies; N-Acc, number of accelerations; PACES-16, 16-item physical activity enjoyment scale; PACES-18, 18-item physical activity enjoyment scale; SSSG, small-sided soccer game; %T, percentage of time; VAS, visual analog scale.

a Higher scores on both the PACES (16- or 18-item versions) and the FUN scale (0-10) indicate a greater perception of enjoyment.^33^

### Internal Load During SSSG

Internal load was assessed by HR response in 15 studies,^1,4,10,13,16,18,19,26,28,29,33,36,38,43,45^ RPE in 10 studies,^1,4,16,18,28,29,33,38,43,45^ and lactate concentration in 1 study.^38^

#### HR Response

The HR response was quantified by beats per minute (bpm) (145-178 bpm), percentage of maximum HR (HRM) (71-88% HRM), percentage of HR reserve (HRR) (75-80% HRR), and percentage of time in HR zones.

Six studies compared the HR response with different types of SSSG.^13,26,28,29,33,45^ The comparisons included variations in the number of players,^28,33^ pitch size,^45^ use of individual marking,^29^ type of regime,^13^ and limitations in the mode of displacement.^26^

According to format type, Póvoas et al^33^ implemented SSSG in 2v2 and 4v4 formats, incorporating both boys and girls in the games, with no significant differences found between formats. On the other hand, Coutinho et al^28^ compared 3v3 and 4v4 formats and found that participants who performed 3v3 spent more time in intensity zones >90% HRM, whereas in 4v4 formats, they spent more time in HR zones <75% and between 85% and 89% HRM.

The effect of pitch size was analyzed only in 1 study,^45^ which compared HR and RPE response in 3v3 SSSG performed on badminton (6.1 × 13.4 m), volleyball (9 × 18 m) and basketball (14.2 × 26.5 m) courts. HR response was higher when using the volleyball and basketball courts than using a badminton court.

One study included the comparison of 2 types of regimen.^13^ A continuous regimen of 16 minutes was implemented and compared with a fractional regimen of 4 sets of 4 minutes and 2 minutes pause. The comparisons showed no differences between regimen types.

Implementing man-marking during the game generated a significant increase in the percentage of the HRR during SSSG.^29^ In addition, restricting the type of movement (walking football) increased the time in zones below 70% of the HRR and decreased the time in intensity above 80% of the HRR (Table 5).^26^

**Table 5.** Effect of structural adjustment and regimen type on workloads and perceived enjoyment during SSSG

		Workload				Perceived enjoyment*^a^*	
		Internal load		External load			
Task adjustment: format							
Póvoas et al^33^	2v2^girls^ (a)	bpm	154.0 ± 21.0			FUN 0-10	5.4 ± 2.3
		% HRM	78.0 ± 9.0				
		%T >80% HRM	38.0 ± 37.0				
		RPE 0-10	3.5 ± 2.5				
	4v4^Girls^ (b)	bpm	150.0 ± 22.0			FUN 0-10	6.7 ± 2.1
		% HRM	75.0 ± 9.0				
		%T >80% HRM	34.0 ± 38.0				
		RPE 0-10	6.3 ± 2.9 (>a)				
	2v2^boys^ (c)	bpm	163.0 ± 13.0			FUN 0-10	5.4 ± 3.3
		% HRM	81.0 ± 6.0				
		%T >80% HRM	55.0 ± 25.0				
		RPE 0-10	5.4 ± 2.5				
	4v4^boys^ (d)	bpm	159.0 ± 10.0			FUN 0-10	6 ± 2.8
		% HRM	79.0 ± 5.0				
		%T >80% HRM	38.0 ± 32.0				
		RPE 0-10	5.9.0 ± 2.5				
	2v2^all^ (e)	% HRM	78.0 ± 9.0				
		%T >80% HRM	43.0 ± 33.0				
		RPE 0-10	5.5.0 ± 2.5 .0				
	4v4^all^ (f)	% HRM	76.0 ± 9.0				
		%T >80% HRM	39.0 ± 32.0				
		RPE 0-10	5.5 ± 2.7				
Coutinho et al^28^	3v3 (a)	%T <75% HRM	14.3 ± 13.3				
		%T 75-84.9% HRM	15.3 ± 29.5				
		%T 85-89.9% HRM	11.3 ± 11.0				
		%T >90% HRM	49.0 ± 42.3 (>b)				
		RPE 6-20	14.6 ± 0.8 (>b)				
	4v4 (b)	%T <75% HRM	28 ± 31.8 (>a)				
		%T 75-84.9% HRM	22.5 ± 22.5				
		%T 85-89.9% HRM	20.8 ± 27.8 (>a)				
		%T >90% HRM	29.0 ± 38.5				
		RPE 6-20	14.1 ± 0.7				
Type of regime: fractional SSSG and continuous SSSG							
Gómez-Álvarez et al^13^	F-SSSG (a)	bpm	178.4 ± 8.1			PACES-16	33.7 ± 6.2
		% HRM	88.5 ± 4.4				
	C-SSSG (b)	bpm	177.2 ± 9.7			PACES-16	33.2 ± 5.8
		% HRM	88.6 ± 4.8				
Task adjustment: pitch configuration							
Toh et al^45^	6.1 × 13.4 Badminton (a)	% HRM	76.0 ± 8.5	Counts	2436.86 ± 444.48	PACES-16	58.7 ± 10.1
		%T <70% HRM	28.4 ± 30.3				
		%T 70-80% HRM	35.5 ± 18.6				
		%T 80-90% HRM	31. ± 29.7				
		%T 90-95% HRM	3.8 ± 9.3				
		%T 95-100% HRM	0.8 ± 3.8				
		RPE 6-20	13.0 ± 0.5				
	9 × 18 Volleyball (b)	% HRM	82.0 ± 8.8 (>a)	Counts	2607.94 ± 634.97	PACES-16	63.7 ± 7.3
		%T <70% HRM	11.1 ± 14.7 (<a)				
		%T 70-80% HRM	34.3 ± 30.8 (>a)				
		%T 80-90% HRM	29.3 ± 23.5 (>a)				
		%T 90-95% HRM	16.7 ± 80.2 (>a)				
		%T 95-100% HRM	7.9 ± 15.5 (>a)				
		RPE 6-20	12.9 ± 0.4				
	14.2 × 26.5 Basketball (c)	% HRM	82.0 ± 9.4 (>a)	Counts	2797.35 ± 558.00 (>a-b)	PACES-16	65.9 ± 7.3
		%T <70% HRM	14.4 ± 15.9 (<a)				
		%T 70-80% HRM	32.5 ± 27.6 (>a)				
		%T 80-90% HRM	30.1 ± 22.5 (>a)				
		%T 90-95% HRM	17.6 ± 19.7 (>a)				
		%T 95-100% HRM	5.9 ± 12.1 (>a)				
		RPE 6-20	12.1 ± 1.63				
Task adjustment: scoring method, tactical mission, and action restriction							
Ngo et al^29^	MM-G (a)	%HRR	80.5 ± 5.8 (>b)				
		RPE	7.1 ± 0.7 (>b)				
	NMM-G (b)	%HRR	75.7 ± 4.7				
		RPE	6.0 ± 0.9				
	MM-NG (c)	%HRR	80.5 ± 4.1 (>d)				
		RPE	7.4 ± 0.8				
	NMM-NG (d)	%HRR	76.1 ± 4.2				
		RPE	6.9 ± 0.8				
Lind et al^26^	Normal SSG (a)	%T <60% HRR	8.7 ± 3.8			PACES-18	4.2 ± 0.21
		%T 60-70% HRR	14.8 ± 5.9				
		%T 70-80% HRR	23.72 ± 4.85				
		%T 80-90% HRR	35.9 ± 6.9 (>b)				
		%T >90% HRR	15.8 ± 7.1 (>b)				
	Walking SSSG (b)	%T <60% HRR	49.8 ± 66.6 (>a)			PACES-18	4.1 ± 1
		%T 60-70% HRR	27.9 ± 42.6 (>a)				
		%T 70-80% HRR	12.9 ± 32.8				
		%T 80-90% HRR	6.8 ± 33.9				
		%T >90% HRR	1.8 ± 18.6				

bpm, beats per minute; HR, heartrate; %HRM, percentage of maximum HR; %HRR, percentage of the reserve HR; MM-G, man-marking with goal; MM-NG, man-marking without goal; NMM-G, no man-marking with goal; NMM-NG, no man-marking without goal; PACES-16, 16-item physical activity enjoyment scale; PACES-18, 18-item physical activity enjoyment scale; RPE, rate perception exertion; SSSG, small-sided soccer game; %T, percentage of time.

a Higher scores on both the PACES (16- or 18-item versions) and the FUN scale (0-10) indicate a greater perception of enjoyment.

Studies analyzing the effect of contextual factors found that verbal encouragement by the trainer increased HR response and RPE during SSSG,^19,38^ whereas contradictory results were reported regarding the effect of age and sex.^1,10,33,36^ In addition, HR response did not differ according to body mass index or school sports participation (Table 6).^1^

**Table 6.** Influence of contextual factors on the internal and external burden during SSSG

		Workload				Perceived enjoyment*^a^*	
		Internal load		External load			
Teacher/coach participation							
Sahli et al^38^	Encouragement (a)	% HRM	88.06 ± 2.25 (>b)			PACES-18	98.9 ± 4.9 (>b)
		Lactate	4.43 ± 0.91(>b)				
		RPE 0-10	7.62 ± 0.79 (>b)				
	No encouragement (b)	% HRM	84.31 ± 2.2			PACES-18	91.5 ± 5.4
		Lactate	3.62 ± 0.6				
		RPE 0-10	6.62 ± 0.81				
Hammami et al^19^	Encouragement (a)	Bpm	168.9 ± 9.3 (>b)				
	No encouragement (b)	bpm	145.93 ± 5.81				
Age of participants							
Randers et al^36^	9 years (a)	%HRM	80.4 ± 7.49				
		bpm	156 ± 18.73				
		%T <70% HRM	25.1 ± 21.42				
		%T 70-80% HRM	25.27 ± 14.24				
		%T 80-90% HRM	31.95 ± 15.30				
		%T 90-95% HRM	12.55 ± 13.24				
		%T 95-100% HRM	4.89 ± 5.25				
	12 years (b)	%HRM	79.5 ± 7.20				
		bpm	153 ± 12				
		%T <70% HRM	22.98 ± 24.48				
		%T 70-80% HRM	21.68 ± 13.68				
		%T 80-90% HRM	36.84 ± 17.64				
		%T 90-95% HRM	12.88 ± 10.74				
		%T 95-100% HRM	5.05 ± 5.88				
Evangelio et al^10^	Third grade (a)	bpm	166 ± 22.9	Max-speed	8.8 ± 1.5		
				AC -1.99; -1.00	20.8 ± 8.3 (>b-d)		
				AC -0.99; -0.50	59.4 ± 15.9 (>b-d)		
				AC 0.50; 0.99	60.1 ± 16.3 (>b-d)		
				AC 1; 1.99	19 ± 8.3 (>b)		
	Fourth grade (b)	bpm	147.9 ± 16.4	Max-speed	8 ± 2		
				AC -1.99; -1.00	11.3 ± 6.8		
				AC -0.99; -0.50	41.2 ± 9.5		
				AC 0.50; 0.99	38.9 ± 1.1		
				AC 1; 1.99	11.4 ± 8.3		
	Fifth grade (c)	bpm	159.9 ± 13.5	Max-speed	10 ± 3.6		
				AC -1.99; -1.00	20.9 ± 7.1 (>b)		
				AC -0.99; -0.50	57.3 ± 10.7 (>b)		
				AC 0.50; 0.99	56.7 ± 7.9 (>b)		
				AC 1; 1.99	17.7 ± 5.8		
	Sixth grade (d)	bpm	174.3 ± 16.4 (>a-b)	Max-speed	12.2 ± 4.4 (>a-b)		
				AC -1.99; -1.00	17.6 ± 4.8		
				AC -0.99; -0.50	44.3 ± 10.8		
				AC 0.50; 0.99	45.2 ± 10.3		
				AC 1; 1.99	14.3 ± 2.5		
Sex of participants							
Bendiksen et al^1^	Boys (a)	bpm	158 ± 18				
		%HRM	78 ± 8				
		%T>90% HRM	13 ± 17				
	Girls (b)	bpm	150 ± 17				
		%HRM	74 ± 8				
		%T>90% HRM	10 ± 16				
Póvoas et al^33^	Boys (a)	%HRM	77 ± 4 (>d)			FUN 0-10	6.3 ± 2.3 (>c)
	Girls (b)	%HRM	77 ± 7 (>d)			FUN 0-10	6.5 ± 1.9 (>c)
	Boys vs mix (c)	%HRM	74 ± 7 (>d)			FUN 0-10	4.4 ± 2.3
	Girls vs mix (d)	%HRM	71 ± 10			FUN 0-10	6.6 ± 1.9 (>c)
Nutritional status							
Bendiksen et al^1^	BMI <17.5	bpm	160 ± 17				
		%HRM	77 ± 9				
		%T>90% HRM	14 ± 21				
	BMI >17.5	bpm	150 ± 19				
		%HRM	74 ± 9				
		%T>90% HRM	11 ± 13				
Participation in formal sport							
Bendiksen et al^1^	Actives	bpm	158 ± 17				
		%HRM	77 ± 8				
		%T>90% HRM	13 ± 17				
	Inactives	bpm	153 ± 20				
		%HRM	74 ± 9				
		%T>90% HRM	11 ± 19				

AC, acceleration (m/s^2^); bpm, beats per minute; HR, heartrate; %HRM, Percentage of Maximum HR; %HRR, Percentage of the Reserve HR; Max-speed, maximum speed (km/h); MM-G, man-marking with goal; MM-NG, man-marking without goal; NMM-G, no man-marking with goal; NMM-NG, no man-marking without goal; PACES-16, 16-item physical activity enjoyment scale; PACES-18, 18-item physical activity enjoyment scale; RPE, rate perception exertion; %T, percentage of time; SSSG, small-sided soccer game.

a Higher scores on both the PACES (16- or 18-item versions) and the FUN scale (0-10) indicate a greater perception of enjoyment.

Randers et al^36^ compared the HR response during 3v3 SSSG in 9-year-old children (court size: 9 × 7 m) versus 12-year-old children (court size: 20 × 13 m) without finding significant differences in bpm, HRM percentage, or time in each HR zone, whereas Evangelio et al^10^ compared HR during SSSG between third (8.96 ± 0.43 years), fourth (10.10 ± 0.63 years), fifth (10.91 ± 0.28), and sixth (12.23 ± 0.46) graders, finding that sixth graders had higher HR responses compared with third and fourth graders.

The effect of sex was analyzed by Bendiksen et al^1^ during SSSG 3v3, without finding differences in bpm, HRM percentage, or time >90% of HRM. Contrarily, Póvoas et al^33^ reported a lower HR response in girls when playing together with boys than when playing among girls only.

#### Perceived Exertion

RPE was assessed using the Borg rating of perceived exertion scale (Borg scale of 0-10 or 6-20), and the Children’s OMNI Perceived Exertion (OMNI Scale of 0-10). The overall results showed that the RPE ranged between 3 to 7 out of 10 and 12 to 15 out of 20 for the Borg scale (Table 3).

Changes in RPE according to the format were reported by 2 studies.^28,33^ Póvoas et al^33^ compared 2v2 and 4v4 without finding differences in RPE; however, when performing analysis by sex, they reported a higher RPE in 4v4 in girls. In addition, Coutinho et al,^28^ in a study that included only boys, noted that the 3v3 format generated a higher RPE than 4v4.

Comparisons in RPE according to pitch size reported no significant differences,^45^ but increases in RPE were reported with the implementation of man-marking and verbal motivation of the coach.^19,31,38^

#### Blood Lactate

Capillary blood lactate concentration immediately after SSSG was assessed in only 1 study by Sahli et al,^38^ where it was reported that verbal encouragement during SSSG increased lactate concentration compared with an SSSG without verbal encouragement.

### External Load During SSSG

External loading was evaluated in 3 studies,^10,43,45^ by measuring total distance traveled,^43^ average speed,^43^ maximum speed,^10^ time per each speed zone,^43^ and number of accelerations (Table 3).^10,45^

The effect of the pitch size on the magnitude of acceleration was assessed by Toh et al.^45^ Their results showed that a 3v3 SSSG performed on the dimensions of a basketball pitch generates higher magnitude accelerations than playing in badminton or volleyball pitches.

Evangelio et al^10^ compared the maximum velocity and number of accelerations during SSSG in third (8.96 ± 0.43 years), fourth (10.10 ± 0.63 years), fifth (10.91 ± 0.28), and sixth (12.23 ± 0.46) graders. Their results showed that third graders performed more decelerations from −1.99 m/s^2^ to −1 m/s^2^ and −0.99 m/s^2^ to −0.50 m/s^2^ and more accelerations between 0.50 m/s^2^ and 0.99 m/s^2^ than fourth and sixth graders and more accelerations from 1 m/s^2^ to 1.99 m/s^2^ than fourth graders. Similarly, fifth graders performed more decelerations ranging between −.99 m/s^2^ to 1 m/s^2^ and −0.99 m/s^2^ to 0.50 m/s^2^ and more accelerations between 0.50 m/s^2^ to 0.99 m/s^2^ than fourth graders. In addition, sixth graders reached higher maximum velocity than third and fourth graders.

### Enjoyment Perception During SSSG

Enjoyment perception was assessed in 6 studies using the PACES scale with 16 and 18 items,^13,16,26,38,45^ or a visual analogical FUN scale 0-10,^33^ where higher scores indicate greater enjoyment. Both methods of assessing perceived enjoyment were conducted immediately after the conclusion of each SSSG. The overall results showed scores between 33.2 to 65.9 and 75.6 to 98.9 for the 16- and 18-item PACES scale, respectively, whereas for the FUN scale, scores ranged between 4.4 and 7.3 (Table 3).

Studies comparing perceived enjoyment by number of players,^33^ pitch size,^45^ or regime type reported no differences.^13^ Performing SSSG with verbal motivation from the trainer generated a higher perception of enjoyment.^38^ In addition, performing SSSG with mixed teams of boys and girls resulted in lower enjoyment perception.^33^

## Discussion

This systematic review aimed to describe the workload and perceived enjoyment associated with SSSG in children and adolescents and to determine how different variations of the game structure and contextual factors affect SSSG-associated workload and enjoyment perception. The main findings include: (1) internal load measures primarily used HR (145-175 bpm; 71-88% HRM) and perceived exertion (RPE 3-7 or 12-15, depending on the Borg scale variant); (2) limited external load evidence, with only 3 studies reporting distance, speed, or accelerations; (3) SSSG are characterized by a high perception of enjoyment; and (4) evidence on SSSG prescription variables or contextual factors is limited, although there is some evidence that formats with a small number of players, larger pitches, and man-marking tend to generate greater internal and external loads. There is also evidence that verbal encouragement promotes greater internal loads during SSSG.

### Workload During SSSG for Untrained Children and Adolescents

All studies included reported internal load measures, while only 3 assessed external loads during SSSG.^10,43,45^ Internal load monitoring was achieved mainly through HR and RPE, with 1 study using capillary blood lactate concentration. External load measures relied on time-motion analyses.^10,43,45^ It is likely that the incipient incorporation of SSSG in exercise programs in the untrained population has led to prioritizing the use of simple-to-use and low-cost measures of intensity. However, although they are valid indicators of intensity and used widely in soccer,^21^ it has been described that HR, RPE, and blood lactate could overestimate or underestimate intensity during SSSG depending on affective responses, intermittent actions or high intensity and short duration actions occurring during the game.^21,42^ On the other hand, external load monitoring, although more complex and expensive, provides complementary information that assists in understanding the metabolic and mechanical demands of SSSG. Future research should incorporate external load technologies such as GPS to provide objective metrics, including distance, speed, and energy expenditure, to better understand and optimize the intensity and benefits of SSSG-based training for health promotion.^21,35^

Characterization of the intensity profile is a essential components of exercise prescription, and it has been proposed that moderate-to-vigorous-intensities are associated with more health benefits in children and adolescents.^3^ Our review indicates that SSSG often achieve HR levels >64% HRM, 40% HRR, or RPE scores exceeding 5 out of 10 or 12 to 13 out of 20.^6,34^ Our results showed that, on average, SSSG are classified as moderate intensity (77% HRM, RPE 5 out of 10, or 13 out of 20), although 50% of studies reported vigorous intensity (>77% HRM). Internal loading during SSSG varies considerably, influenced by game structure, regimen, and contextual factors.^5,21,40^ In addition, 7 studies analyzed time in different HR zones,^1,4,26,28,36,45^; however, inconsistencies in defining these zones highlight a lack of standardization, complicating interpretation.^23,24^

Nevertheless, it was apparent that, during SSSG, participants could spend >50% of the game at a vigorous intensity (>77% HRM or 60% HRR; RPE 7 out of 10 or 14-17 out of 20). For example, 40% of playtime occurred >80% HRM, ~30% between 80% and 90% HRM, and ~20% >90% HRM. This high-intensity cumulative time aligns with evidence linking 10-minute increments of vigorous exercise to improved cardiometabolic health,^44^ body composition, and physical fitness benefits.^14^

On the other hand, although RPE measures have been validated, we identified inconsistencies compared with the results derived from HR. While HR data classified SSSG as moderate-to-vigorous intensity, RPE ranged from light to vigorous. Higher perceived enjoyment can explained these differences in results, leading to lower RPE scores during SSSG.^42^ This response in the RPE could be relevant for developing high-intensity training programs because high intensity exercise can trigger negative affective responses, which, consequently, can compromise training adherence and compliance.^9^ Indeed, some studies have compared high-intensity interval training (HIIT) and SSSG, reporting similar intensities between SSSG and HIIT,^42^ as well as similar long-term effects on fitness.^7^ Only a few studies included in this systematic review were structured similarly to HIIT. For example, Sahli et al^38^ implemented an SSSG with 4 sets of 4 minutes and 3 minutes rest and reported mean intensities of 88% HRM, RPE 7/ out of 10, and blood lactate concentration of 4 mmol/l, representing vigorous intensity.

Our results also suggest that there is a partial understanding of how SSSG can be prescribed to control training load. Analysis of the effect of manipulating regimen type, structural, and contextual variables was reported in only 6 studies. The manipulation of these variables has been the focus of SSSG research in the trained population since this information is key to prescribe training based on SSSG for optimization of training loads according to the planned objective.^2,5,21,40^ Our results suggest that a smaller number of players,^28,33^ larger pitch sizes,^45^ and assigning man-marking,^29^ increased internal loads, which is consistent with previous reviews carried out in a trained population.^5,21^ However, characteristics such as sex and age have shown inconsistent results.

In addition, this review reported limited evidence regarding external loads associated with SSSG.^10,43,45^ The characterization of the external load, in addition to providing information on the stress profile, is associated with risk of injury.^35^ For example, acceleration and deceleration leads to high levels of mechanical stress in joints, muscles, and tendons, which can negatively affect the musculoskeletal system.^47^ In particular, decelerations have been proposed to induce neuromuscular fatigue and tissue damage.^20^

### Enjoyment Perception During SSSG for Untrained Children and Adolescents

Our results of perceived enjoyment suggest that SSSG could be a good strategy for developing motivational programs that lead to good adherence and fidelity.^13,16,26,33,38,45^ This is probably related to soccer’s joyful, social, and popular components, which may favor a smoother integration of SSSGs into children and adolescents daily interests and activities. For example, large-scale studies have included SSSG in the “11 for health in Denmark” exercise school program that included 3127 boys and girls aged 10 to 12 years. Their results showed that, after 11 weeks of intervention, participants improved their physical fitness and health measures and reported moderate-to-high enjoyment scores.^25^ Notably, the results of this review suggest that the perception of enjoyment is increased by verbal encouragement of the teacher/coach,^38^ and may be decreased by mixed-sex SSSG.^33^ The effect of verbal encouragement is consistent with that reported in the trained population, whereas the impact of team composition according to sex has been less studied. Póvoas et al^33^ found no differences in enjoyment when SSSG was performed between participants of the same sex; however, when a team composed of boys played against a mixed boys and girls team, they reported a lower perception of enjoyment. This is consistent with the results of the “11 for Health in Denmark” program, in which girls reported a lower perception of enjoyment, which could be explained by sex-specific sports preferences.^8^ The structural aspects of SSSG were also evaluated in 3 studies,^26,33,45^ which identified that pitch size,^45^ format,^33^ and restriction of locomotion type during SSSG did not affect perceived enjoyment.^26^

### Practical Implications

This systematic review identifies key practical implications for implementing SSSG across various contexts, including schools, community centers, and health promotion programs. SSSG demonstrate flexibility and adaptability, making them suitable for diverse spaces and participant needs. SSSG intensity can be effectively tailored through structural modifications, such as reducing player numbers, increasing pitch size, or employing man-marking strategies, with smaller teams and larger pitches shown to increase internal and external loads. In addition, SSSG foster high levels of motivation and adherence due to their enjoyable and engaging nature, particularly among pediatric and untrained populations, as they are associated with low perceived exertion. For educators and coaches, SSSG provide a practical tool for prescribing moderate-to-vigorous intensity exercise, with verbal encouragement and sex-specific team composition enhancing both intensity and enjoyment. SSSG are particularly valuable in health programs aiming to promote physical activity and improve fitness outcomes in children and adolescents.

### Limitations and Recommendations for Future Research

The following limitations were identified in this systematic review: (1) there is a low volume of research adequacy reporting the workload and enjoyment in SSSG interventions; (2) small sample sizes and lack of standardized measures across studies limit the generalizability of the findings of this systematic review. For example, the use of variable HR zones and different RPE scales makes direct comparisons between studies difficult and complicates the development of standardized recommendations for prescribing SSSG in untrained children and adolescents; (3) only 3 studies analyzed external loads, so the descriptions of these measures are limited to a few SSSG designs; (4) only 5 studies analyzed structural aspects, 1 analyzed the type regime, and only 6 analyzed contextual factors, which does not allow drawing robust conclusions regarding the effect of these dimensions on SSSG intensity and enjoyment; and (5) exclusion of databases due to institutional access restrictions (e.g., Embase, CENTRAL, or CINAHL) may have introduced database bias, limiting the completeness of the literature search and potentially omitting relevant studies.

## Conclusion

SSSG, as a training strategy for untrained children and adolescents, still requires further research. Current evidence suggests that SSSG elicit moderate-to-vigorous internal loads, with HRs ranging from 71% to 88% HRM and RPE values between 3 to 7 out of 10 or 12 to 15 out of 20, along with high levels of perceived enjoyment. The evidence on external loads is still limited, hindering adequate characterization.

Current results suggest that smaller number of players, larger pitch size, and man-marking result in higher workloads. Verbal encouragement appears to enhance both internal loads and enjoyment, while mixed-sex SSSG may reduce them. Furthermore, enjoyment in SSSG needs to be investigated as a strategy to maximize the long-term effectiveness of health promotion programs and in structured training environments.

## Supplemental Material

sj-docx-1-sph-10.1177_19417381251385590 – Supplemental material for Workload and Enjoyment Perception in Small-Sided Soccer Games: A Systematic Review of Studies in Untrained Children and Adolescents Supplemental material, sj-docx-1-sph-10.1177_19417381251385590 for Workload and Enjoyment Perception in Small-Sided Soccer Games: A Systematic Review of Studies in Untrained Children and Adolescents by Nicolás Gómez-Álvarez, Leonel Federico-Tuccelli, Paula SanMartín-Godoy, Mario Vieyra-Fuenzalida, Felipe Hermosilla-Palma, Tomás Reyes-Amigo, José Oliveira and Hélder Fonseca in Sports Health

sj-docx-2-sph-10.1177_19417381251385590 – Supplemental material for Workload and Enjoyment Perception in Small-Sided Soccer Games: A Systematic Review of Studies in Untrained Children and Adolescents Supplemental material, sj-docx-2-sph-10.1177_19417381251385590 for Workload and Enjoyment Perception in Small-Sided Soccer Games: A Systematic Review of Studies in Untrained Children and Adolescents by Nicolás Gómez-Álvarez, Leonel Federico-Tuccelli, Paula SanMartín-Godoy, Mario Vieyra-Fuenzalida, Felipe Hermosilla-Palma, Tomás Reyes-Amigo, José Oliveira and Hélder Fonseca in Sports Health
