# Supplementary material for: Workload and Enjoyment Perception in Small-Sided Soccer Games: A Systematic Review of Studies in Untrained Children and Adolescents
Source: Sports Health. 2025 Oct 31;18(2):250–68. doi: 10.1177/19417381251385590 (PMC12578626; doi:10.1177/19417381251385590)
Supplement: Supplementary file 1 [file sj-docx-1-sph-10.1177_19417381251385590.docx]

**Supplementary file 1:**

**Title:** Workload and enjoyment perception in small-sided soccer games: A systematic review of studies in untrained children and adolescents

**Full details of search terms, search algorithm, dates and databases with number of hits per database provided.**

| Databases | **Search terms and search algorithm** | **Number** |
| --- | --- | --- |
| PubMed | (((children[Title/Abstract] OR Childhood[Title/Abstract] OR adolescent[Title/Abstract] OR youth[Title/Abstract] OR young[Title/Abstract]) AND (small-sided games[Title/Abstract] OR small-sided soccer games[Title/Abstract] OR SSG[Title/Abstract] OR SSSG[Title/Abstract])) AND (Soccer[Title/Abstract] OR football[Title/Abstract])) AND (workload[Title/Abstract] OR response[Title/Abstract] OR demands[Title/Abstract] OR "acute effects"[Title/Abstract] OR load[Title/Abstract] OR physiological[Title/Abstract] OR intensity[Title/Abstract] OR "heart rate"[Title/Abstract] OR lactate[Title/Abstract] OR enjoyment[Title/Abstract] OR velocity[Title/Abstract] OR acceleration[Title/Abstract] OR deceleration[Title/Abstract]) | 148 |
| Web of Science | children OR Childhood OR adolescent OR youth OR young (Topic) AND small-sided games OR small-sided soccer games OR SSG OR SSSG (Topic) AND soccer OR Football (Topic) AND workload OR response OR demands OR "acute effects" OR load OR physiological OR intensity OR "heart rate" OR lactate OR enjoyment OR velocity OR acceleration OR deceleration (Topic) | 401 |
| Scopus | ( TITLE-ABS-KEY ( children OR childhood OR adolescent OR youth OR young ) AND TITLE-ABS-KEY ( small-sided AND games OR small-sided AND soccer AND games OR ssg OR sssg ) AND TITLE-ABS-KEY ( soccer OR football ) AND TITLE-ABS-KEY ( workload OR response OR demands OR "acute effects" OR load OR physiological OR intensity OR "heart rate" OR lactate OR enjoyment OR velocity OR acceleration OR deceleration ) ) | 338 |
| Scielo (WoS) | children OR Childhood OR adolescent OR youth OR young (Topic) AND small-sided games OR small-sided soccer games OR SSG OR SSSG (Topic) AND soccer OR Football (Topic) AND workload OR response OR demands OR "acute effects" OR load OR physiological OR intensity OR "heart rate" OR lactate OR enjoyment OR velocity OR acceleration OR deceleration (Topic) | 11 |
